# Multilocus Bayesian Estimates of Intra-Oceanic Genetic Differentiation, Connectivity, and Admixture in Atlantic Swordfish (*Xiphias gladius * L.)

**DOI:** 10.1371/journal.pone.0127979

**Published:** 2015-06-09

**Authors:** Brad L. Smith, Ching-Ping Lu, Blanca García-Cortés, Jordi Viñas, Shean-Ya Yeh, Jaime R. Alvarado Bremer

**Affiliations:** 1 Department of Marine Biology, Texas A&M University at Galveston, OCSB 3029, Galveston, TX 77553, United States of America; 2 Department of Wildlife and Fisheries Sciences, Texas A&M University, 210 Nagle Hall, TAMU 2258, College Station, TX, 75044, United States of America; 3 Centro Oceanográphico A Coruña, Instituto Español de Oceanografia, Muelle de las Animas s/n P.O. Box 130, 15080 A Coruña, Spain; 4 Department de Biologia, Universitat de Girona, Campus Montilivi, E-17071 Girona, Spain; 5 Institute of Oceanography, National Taiwan University, Taipei 106, Taiwan; Tuscia University, ITALY

## Abstract

Previous genetic studies of Atlantic swordfish (*Xiphias gladius* L.) revealed significant differentiation among Mediterranean, North Atlantic and South Atlantic populations using both mitochondrial and nuclear DNA data. However, limitations in geographic sampling coverage, and the use of single loci, precluded an accurate placement of boundaries and of estimates of admixture. In this study, we present multilocus analyses of 26 single nucleotide polymorphisms (SNPs) within 10 nuclear genes to estimate population differentiation and admixture based on the characterization of 774 individuals representing North Atlantic, South Atlantic, and Mediterranean swordfish populations. Pairwise *F*_ST_ values, AMOVA, PCoA, and Bayesian individual assignments support the differentiation of swordfish inhabiting these three basins, but not the current placement of the boundaries that separate them. Specifically, the range of the South Atlantic population extends beyond 5°N management boundary to 20°N-25°N from 45°W. Likewise the Mediterranean population extends beyond the current management boundary at the Strait of Gibraltar to approximately 10°W. Further, admixture zones, characterized by asymmetric contributions of adjacent populations within samples, are confined to the Northeast Atlantic. While South Atlantic and Mediterranean migrants were identified within these Northeast Atlantic admixture zones no North Atlantic migrants were identified respectively in these two neighboring basins. Owing to both, the characterization of larger number of loci and a more ample spatial sampling coverage, it was possible to provide a finer resolution of the boundaries separating Atlantic swordfish populations than previous studies. Finally, the patterns of population structure and admixture are discussed in the light of the reproductive biology, the known patterns of dispersal, and oceanographic features that may act as barriers to gene flow to Atlantic swordfish.

## Introduction

The epipelagic realm of the world’s oceans is a relatively homogeneous and contiguous environment characterized by the near absence of physical barriers. These features when coupled with the high dispersal behavior and demographic characteristics of many marine species result in high levels of gene flow [1]. Specifically, many epipelagic species of fishes are characterized by long distance potential dispersal of pelagic eggs and larvae facilitated by currents, high vagility of juveniles and adults, opportunistic feeding behaviors, eurythermal physiologies of adults, and large effective population sizes. Consequently, the levels of genetic differentiation in marine fishes are typically substantially lower compared to populations of anadromous and freshwater fishes [2]. It is thus not surprising that the distributions of many species of tunas, billfishes, dolphinfishes, lamnid sharks, are cosmopolitan [3, 4], with some species, such as skipjack tuna (*Katsuwonus pelamis*) displaying no genetic differentiation among-oceans [5], and others, like wahoo (*Acanthocybium solandri*) showing only incipient levels inter-oceanic differentiation [6]. Significant inter-oceanic differentiation, however, has been documented in many highly migratory fishes, including bigeye tuna [7], Atlantic bluefin tuna [8, 9], albacore tuna [10, 11], and many species of billfishes [12] and swordfish [13]. Similarly, genetic investigations in several species of billfish have shown intra-oceanic genetic differentiation within the Pacific Ocean, including sailfish [14], white marlin, and blue marlin [12]. Within the Atlantic, no significant population substructure was detected in several epipelagic fishes; including shortfin mako (*Isurus oxyrinchus*) [15], blue marlin (*Makaira nigricans*) and sailfish (*Istiophorus platypterus*) [12], bigeye tuna (*Thunnus obesus*) [16], and in both yellowfin tuna (*Thunnus albacares*) and skipjack tuna [5]. By contrast, swordfish (*Xiphias gladius* L.) shows significant genetic differentiation within the Atlantic Ocean [13, 17, 18] and also within the Mediterranean Sea [19].

Swordfish is a large epipelagic monotypic cosmopolitan species capable of long distance movements, as documented using both conventional tags [20] and pop-up satellite tags (PSATs) [21–24]. Swordfish are subject to intensive commercial exploitation worldwide, and in the Atlantic Ocean they are managed by the International Commission for the Conservation of Tunas (ICCAT) as two separate stocks: the North Atlantic and the South Atlantic separated at 5°N. In turn, North Atlantic swordfish is managed separately from Mediterranean swordfish with a boundary at the Strait of Gibraltar. Previous genetic studies on Atlantic swordfish employing mitochondrial DNA (mtDNA) (see [25] for summary), single copy nuclear DNA (scnDNA) [18, 26, 27], and to lesser exent microsatellite loci [28–30], confirm the differentatiation among North Atlantic, South Atlantic and Mediterranean populations. Some of these studies also suggested that the range of these swordfish populations extend beyond current management boundaries [26, 31]. However, limitations in geographic sampling coverage, and the use of single loci, precluded an accurate placement of boundaries and of estimates of admixture. Multilocus analyses of microsatellite data [29, 30] attempted to refine these estimates by expanding sampling coverage and by utilizing Bayesian individual assignment to identify zones of admixture. While, microsatellite data confirmed the pronounced differences between Atlantic and Mediterranean swordfish [29], it failed to delineate the separation of North Atlantic and South Atlantic swordfish populations.

In recent years there has been an increase in the adoption of single nucleotide polymorphisms (SNPs) as an alternative to microsatellite data in Bayesian analyses of population admixture. In addition to a comparable differentiation power, SNPs have lower associated genotyping errors, simpler mutation models, higher amenability to automation and high throughput technologies, and increased amplification success with low-quality and ancient DNA as compared to microsatellites [32–34]. Further, the levels of genetic differentiation among Atlantic swordfish populations using SNPs was an entire order of magnitude greater compared to microsatellites [35, 36]. Accordingly, in this study we evaluate the patterns of population differentiation and admixture of Atlantic and Mediterranean swordfish based on Bayesian analyses of multilocus nuclear SNPs using a more extensive and representative geographic sampling coverage compared to previous studies. We describe the patterns of differentiation and admixture among North Atlantic, South Atlantic and Mediterranean swordfish populations.

## Materials and Methods

### Sampling

**Fig 1:**
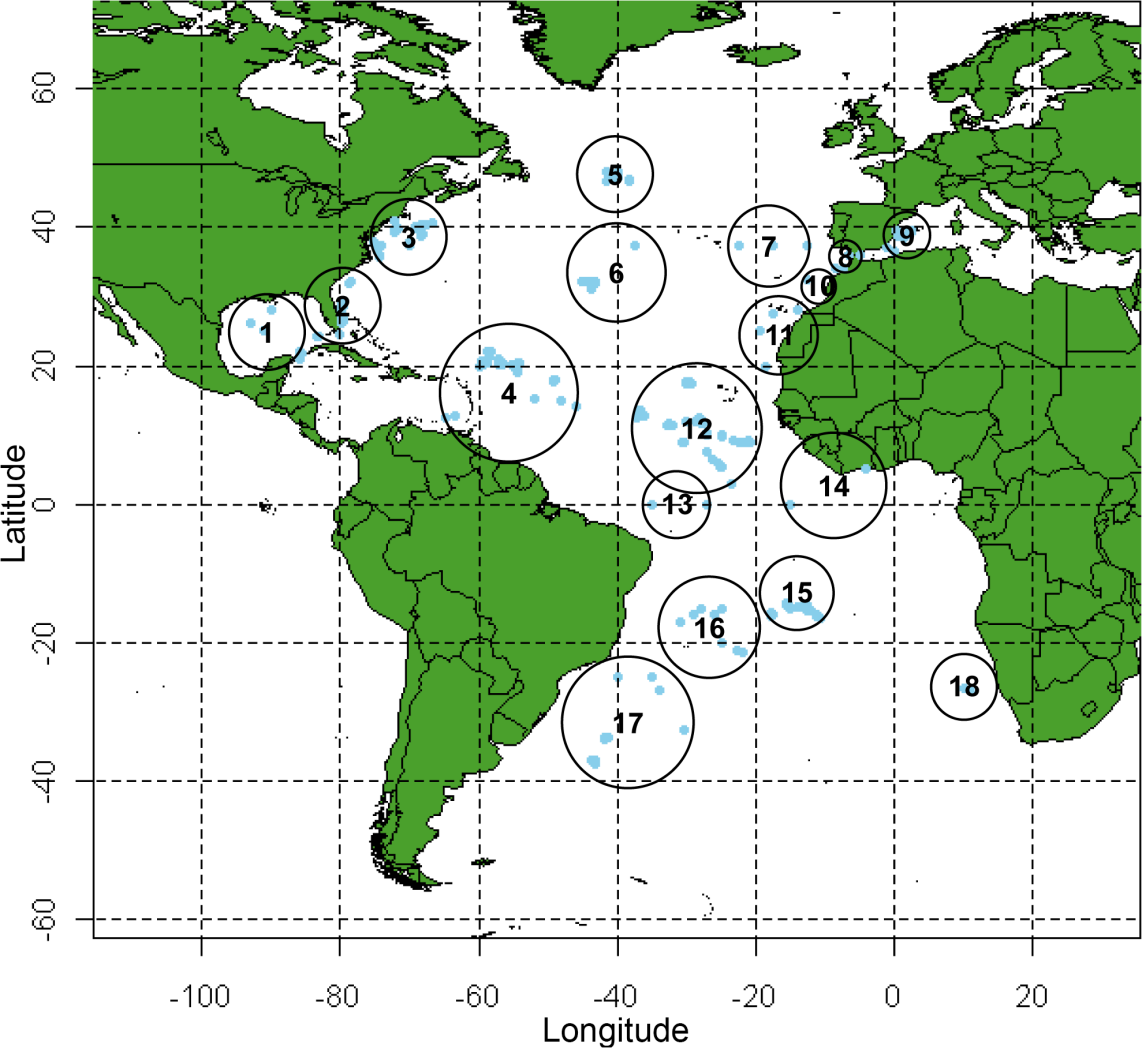
The geographical sampling of 774 swordfish (blue dots) and the locality groupings (numbered black circles) used for analyses as outlined in Table 1.

A total of 774 swordfish specimens from 18 localities in the Atlantic and western Mediterranean were collected from 1991–2006 (Table 1 and Fig 1). Muscle, liver, and heart tissue samples of were collected by observers on commercial longline vessels or obtained from collaborating researchers. Ethical approval was not required for the collection of adult swordfish, as these were collected during routine commercial operations. Swordfish adults were sacrificed by the fishermen by decapitation. In all instances the location of capture for each fish was recorded. Larval swordfish (n = 52), collected as part of icthyoplankton surveys in the northern Gulf of Mexico during the summers of 2005–2006 [37], were also characterized. Permits for fish larvae collections in the Gulf of Mexico were issued by the Highly Migratory Species Management Division of the National Oceanic and Atmospheric Administration.

**Table 1:** Sampling localities for 774 swordfish subdivided into three stocks (North Atlantic, Mediterranean, South Atlantic) by ICATT management boundaries of 5°N and Strait of Gibraltar.

Population	Sample Locality	Latitude	Longitude	Sampling Date(s)	n
North Atlantic					
1	Gulf of Mexico	26-28N	86-93W	06/06-07/06, 06/07	52
2	Florida	21-32N	78-85W	1/93-8/93	49
3	Northeast US	35-42N	67-74W	8/90-11/90, 1/91-3/91	42
4	Lesser Antilles	12-20N	49-64W	10/92-2/93, 2/94, 1/95	48
5	East of Flemish Cap	43-47N	37-41W	9/04-11/04	17
6	Central North Atlantic	32-47N	37-43W	11/91, 12/95, 9/04-11/04	20
7	Iberian	35-40N	10-25W	11/91, 12/92, 3/93-3/93, 12/95-4/96	51
8	Strait of Gibraltar	33-35N	5-10W	8/92, 6/93-9/93, 5/96-11/96	83
10	Morocco	30-33N	12-15W	4/93-5/93, 10/96-11/96, 6/02-8/02	20
11	Western Sahara	20-28N	12-20W	2/92, 5/92, 6/93, 12/95, 4/96, 7/96-11/96	37
12	Cape Verde	5N-17N	21-32W	8/04-12/04, 9/05-10/05	56
Mediterranean					
9	West Mediterranean	35-39N	0-8E	5/92-7/92, 8/03-12/03	59
South Atlantic					
13	Equatorial Brazil	1S-3N	23-35W	11/91, 3/00, 9/04	42
14	Gulf of Guinea	5N	4W	11/91, 7/98-8/98	45
15	Central South Atlantic	14-16S	10-17W	10/04-12/04	28
16	Brazil	15-23S	22-27W	3/96, 5/96, 4/01, 12/04	35
17	Brazil-Uruguay	27-37S	32-42W	8/95-10/95, 4/01, 6/03-7/03	44
18	Namibia	26-27S	10-11E	7/99-8/99	46

(permits: Billfish-SRP-06-01, Billfish-EFP-07-03, and Billfish-EFP-08-03) to Dr. J.R. Rooker. The management boundaries at 5°N, separating the North Atlantic stock from the South Atlantic stock, and at the Strait of Gibraltar, separating the North Atlantic stock from the Mediterranean stock, were initially used to assign localities to the corresponding three management units or stocks currently recognized by ICCAT [38].

### DNA extraction and nuclear loci genotyping

Total genomic DNA was isolated from tissue with a modified TENS and Proteinase K (20 mg/μL) digestion followed by ethanol precipitation without organic extraction [39]. Ten nuclear loci were amplified and genotyped for genetic population structure analyses: acidic ribosomal phosphoprotein P0 (ARP); adenine nucleotide translocator (ANT); aldolase-B (AldB); alpha-skeletal actin (Act2α); ATP synthase beta-subunit (ATPsβ); Calmodulin (*CaM*); Golgi pH regulator (GpHr); lactose dehydrogenase A (ldhA); myosin light chain (Mlc2); and signal recognition particle 54 (SRP54). Details on loci and corresponding SNPs selection, the minimization of ascertainment bias as well as the criteria to design primers and probes that target these, are given in Smith et al. [35]. Briefly, to minimize ascertainment bias potential loci and SNPs were selected after conducting a preliminary characterization with HRMA of 30 swordfish, 10 from each of three geographically disjunct swordfish populations (NW Atlantic, Mediterranean and Pacific). Those amplicons that generated distinct melting profiles, were sequenced, and electropherogram inspections were then used to validate the SNPs responsible for the polymorphic melting curves (see [35] for details). Sequence alignments were also used to evaluate the placement and design of new HRMA-genotyping primers and probes, when required. A total of 85 SNPs were initially identified but only those differing at frequencies >5% among reference samples were targeted for further HRMA primer and or probe design. PCR conditions of selected potential loci were further optimized and validated using additional samples (n = 40) from each of the three reference populations. This process ultimately yielded a total of 26 SNPs among 10 loci that we targeted for genotyping (see Table 2 in [35]). Procedures for the amplification and subsequent genotyping of SNPs for short amplicon high-resolution melting analysis (SA-HRMA) of ARP, ATPsβ, C*aM*, and SRP54 and unlabeled probe high-resolution melting analysis (UP-HRMA) of ANT, GpHR, ldhA, and Mlc2 are described in Smith et al. [35].

Two additional loci, AldB and Act2α, were scored as size polymorphisms as follows. A simple sequence repeat (SSR) identified in AldB [39] was amplified with the following primers; 5’-VIC-TGTGCCCAGTATAAGAAGGATGG-3’ and 5’-CTGTGGAGAATCAGGGCTCC-3’ (JX042447). Polymerase chain reactions (PCR) were conducted in 12.5 μL reactions containing 10 ng of genomic DNA, 1X Econotaq Green Master Mix (Lucigen), and 0.20 μM of each primer. Thermocycling was performed on an Eppendorf Mastercycler (Eppendorf) with an initial denaturation of 10 min at 95°C followed by 35 cycles denaturing for 1 min at 94°C, annealing for 1 min at 54°C, and extension for 1 min at 72°C. PCR products were diluted 1:10 and 1 μL of diluted PCR template and 0.2 μL of ROX size standard (Life Technologies) in 10 μL of Hi-Di formamide were run on an ABI 3130 Genetic Analyzer (Applied Biosystems). SSRs were scored by size and analyzed using GeneMapper v.4.0 (Applied Biosystems).

Locus Act2α contains an SNP that corresponds to a restriction site of endonuclease Hpy8I and was amplified using primers; 5’-GTCACCGGAGTCCAGGACG-3’ and 5’-ATCTGGCACCACACCTTCTACAA-3’ (JX042448) using the same chemistry and thermocycling profile used in AldB amplification. Prior to digestion, the quality and quantity of PCR products were visualized in 1% Tris-acetate (TA) agarose gels via gel electrophoresis prior to digests. Restriction digests were conducted in 10 μL volumes containing 2–4 μL PCR product, 1X Buffer TANGO (Fermentas), and 0.4 U of Hpy8I (Fermentas), that were incubated for 16 hours at 37°C, followed by thermal inactivation at 80°C for 5 min. Restriction Fragment Length Polymorphisms (RFLPs) were separated in 2% TA agarose gels run at 100V for 30 min, and were visualized and captured on a Gel Doc XR (Bio-Rad) UV transilluminator using the Quality One v.4.6 (Bio-Rad) software.

### Data analysis

#### Genetic diversity

The number of alleles, observed (*H*_o_) and expected (*H*_e_) heterozygosities under Hardy-Weinberg equilibrium (HWE), and inbreeding coefficients (*F*_IS_) were calculated using GENALEX version 6.41 [40]. Departures from HWE were estimated using an exact probability test [41, 42] and global linkage disequilibrium using a maximum likelihood-ratio test [43] in GENEPOP version 4.1 (Markov chain parameters: 10000 dememorization steps, 1000 batches, 10000 iterations per batch) [44, 45]. Sequential Bonferroni corrections were applied to adjust the levels of statistical significance for multiple comparisons [46]. An *F*_ST_-outlier detection method, in which observed locus *F*_ST_ values are compared to calculated global *F*_ST_ values expected under neutrality using coalescent simulations [47] was performed in LOSITAN [48]. A total of 50,000 simulations were conducted to detect putative loci under selection. To remove potential selective loci when computing the initial mean *F*_*ST*_, the first simulation used the ‘neutral’ mean *F*_ST_ option with a 0.995 confidence interval, a false discovery rate set at 0.1, and by pooling the sampling localities into the corresponding three populations (K = 3) identified by Bayesian clustering analyses (see below) as follows, North Atlantic (1–7, 10–11), South Atlantic (12–18), and Mediterranean (8–9). The assignment power of each locus was ranked *a posteriori* to Bayesian analysis using the critical population method in WHICHLOCI v1.0 [49] using samples from localities identified as non-mixing areas.

#### Population differentiation

A hierarchical analysis of molecular variance (AMOVA)[50] was implemented in Arlequin version 3.5 [51] to estimate levels of population subdivision. Population differentiation was also evaluated using both global and pairwise *F*_*ST*_ tests [52]. Initially, AMOVA groupings of two (MED and Atlantic) and three (MED, NA, and SA) adhering to the current management regions were evaluated. Alternative groupings in AMOVA were evaluated *a posteriori* to *F*_ST_ and Bayesian analyses (see below). Slatkin’s linearized *F*_ST_ [53] was also calculated in Arlequin. Principal Coordinates Analysis (PCoA) [54], based on a standardized covariance matrix of genetic distances between each locality pair, was carried in GenAlEx version 6.4 [40].

#### Genetic clustering analysis

Genetic population structure and patterns of inter-population gene flow were assessed by Bayesian inference implemented in STRUCTURE version 2.3 [55]. Previous genetic studies using mtDNA and a single nuclear locus identified comparatively low levels of gene flow in between North and South Atlantic swordfish [17, 26, 56]. Accordingly, a no admixture ancestry with correlated allele frequencies model, as outlined in Falush et al. [57] was adopted. Compared to freshwater and anadromous fishes, the populations of marine fishes display weak levels of differentiation (*F*_ST_ < 0.20) [58]. Accordingly, the LOCPRIOR option was implemented to detect weak signals of population structure using an a priori grouping as outlined in Hubisz et al. [59]. The number of clusters (*K*) was estimated using an *ad hoc* approach [55] by obtaining the mean posterior probability of the data (L(*K*)) and the ΔK approach of Evanno et al. [60] using STRUCTURE HARVESTER v0.6.92 [61]. Twenty independent runs for each *K* value (1–10) were performed using 100,000 Markov chain Monte Carlo (MCMC) iterations with a burn-in period of 100,000. Results from STRUCTURE were compared with the results from GENELAND [62], which incorporates individual spatial and genetic data to infer population structure and spatial boundaries between clusters. Individual spatial coordinates were set to the corresponding latitude and longitude of sampling. An uncertainty value on the spatial coordinates of each sample was set to 30 decimal degrees corresponding to the documented displacement of North Atlantic swordfish from tagging experiments [22, 23]. Correlated allele and spatial models were implemented for twenty independent runs with 100,000 MCMC iterations and a burn-in of 10,000 iterations for each *K* value (1–8). After estimating *K*, 25 independent runs with the optimal *K* value were run in GENELAND, 50 independent runs in STRUCTURE, and the optimal alignment of replicates were completed using CLUMPP version 1.1.12 [63].

## Results

### Hardy-Weinberg, linkage disequilibrium, and *F*_ST_ outlier analysis

**Fig 2:**
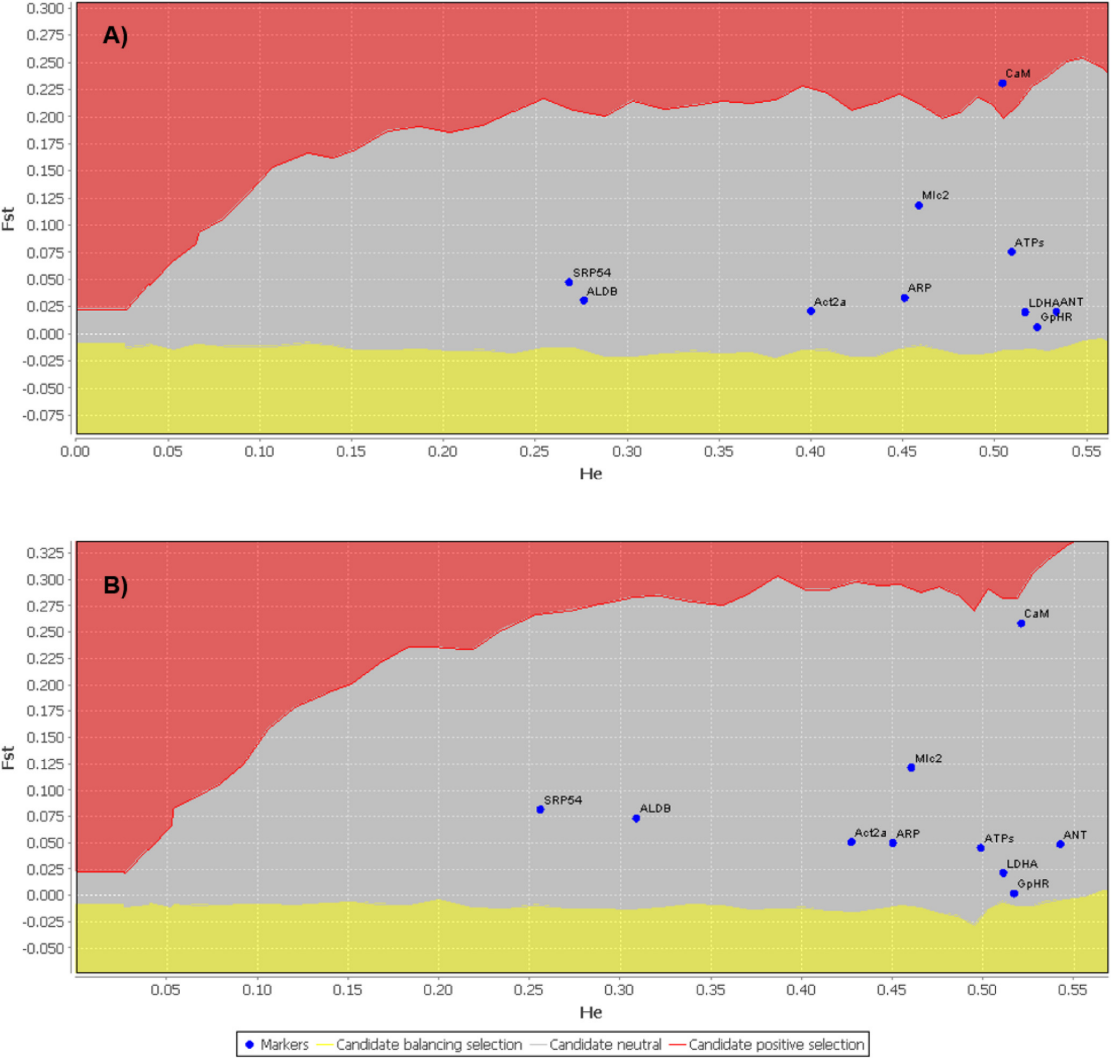
Identification of candidate loci under selection inferred from *F*_ST_ outlier analysis (P < 0.05) [47, 48] of ten nuclear markers using the individuals (A) from all 18 localities and (B) excluding admixture localities and including only localities that correspond to areas of known spawning. *F*_ST_ values for all the loci were notably greater and *H*_e_ for Act2α, AldB, ATPsβ, CaM, and Mlc2 increased when admixed samples were excluded (B) compared to the analysis with all samples (A).

A total of 774 swordfish were genotyped successfully at the ten loci (see supporting S5 Table). Five of these loci contained a single SNP and were bi-allelic and the rest contained both multiple SNPs and alleles. The number of alleles per locus ranged from 2 to 6, and in total 26 SNPs were characterized (see supporting S1 Table). Average (*H*_o_) and expected (*H*_e_) heterozygosities, and inbreeding coefficients (*F*_IS_) are given in Table 2. None of the Fisher’s exact probability tests for departure from HWE were significant (*P* < 0.05) after Bonferroni correction. Genotypic linkage was not significant among the 44 possible pairs of nuclear loci across all samples with (*P* < 0.01) and without (*P* < 0.05) Bonferroni correction. Likewise exact tests for genotypic linkage disequilibrium within each sample were also non-significant after Bonferroni correction (*P* < 0.05) (not shown). Initial *F*_ST_ outlier analysis of the ten loci of all individuals from the 18 localities identified C*aM* as a potentially under selection (*P* < 0.05) (Fig 2A). However, since both STRUCTURE and GENELAND identified several localities (6–7, 10–11) in the North Atlantic as admixture zones there was the possibility that heterozygote deficits biased the outlier analysis. Consequently, this analysis was conducted excluding admixed samples resulting in no locus identified as an outlier (*P* < 0.05). This procedure also yielded higher *F*_ST_ values for all loci as well as higher *H*_e_ values for Act2α, AldB, ATPsβ, *CaM*, and Mlc2. (Fig 2B). The power assignment of each loci using WHICHLOCI v1.0 identified *CaM* as the locus with the highest power of discrimination for North and South Atlantic populations followed by Mlc2, SRP54, AldB, ARP, Act2α, ldhA, GpHR, ATPsβ, and ANT respectively (see supporting S2 Table). Interestingly, *CaM* displayed the lowest power of discrimination between North Atlantic and Mediterranean populations, with Mlc2 yielding the highest power of discrimination between them followed by ATPsβ, Act2α, ldhA, ANT, AldB, SRP54, ARP, and GpHR, respectively (see supporting S3 Table).

**Table 2:** Average genetic diversity of 10 nuclear loci within 18 swordfish populations.

		Locus									
Population		ARP	ALDB	GpHR	Act2α	ATPsβ	CaM	Mlc2	SRP54	LDHA	ANT
1	*A*	2	4	5	2	2	2	3	2	3	5
	*H*_*o*_	0.288	0.288	0.615	0.231	0.500	0.346	0.173	0.327	0.385	0.442
	*H*_*E*_	0.447	0.287	0.581	0.473	0.453	0.497	0.206	0.322	0.450	0.567
	*F*_*IS*_	0.354	-0.005	-0.059	0.513	-0.105	0.304	0.161	-0.014	0.145	0.220
2	*A*	2	4	5	2	2	2	3	2	3	5
	*H*_*o*_	0.347	0.224	0.571	0.592	0.469	0.510	0.265	0.388	0.633	0.571
	*H*_*E*_	0.479	0.205	0.512	0.500	0.493	0.497	0.320	0.359	0.468	0.522
	*F*_*IS*_	0.276	-0.097	-0.115	-0.184	0.047	-0.027	0.170	-0.079	-0.352	-0.094
3	*A*	2	4	5	2	2	2	3	2	3	5
	*Ho*	0.381	0.238	0.571	0.429	0.429	0.476	0.357	0.310	0.476	0.405
	*HE*	0.472	0.218	0.521	0.459	0.427	0.499	0.381	0.262	0.447	0.504
	*FIS*	0.192	-0.094	-0.097	0.067	-0.003	0.045	0.062	-0.183	-0.065	0.197
4	*A*	2	4	5	2	2	2	3	2	3	6
	*H*_*o*_	0.417	0.188	0.521	0.458	0.458	0.500	0.458	0.292	0.583	0.542
	*H*_*E*_	0.444	0.209	0.513	0.413	0.457	0.499	0.400	0.330	0.476	0.545
	*F*_*IS*_	0.062	0.101	-0.015	-0.109	-0.002	-0.002	-0.147	0.116	-0.225	0.007
5	*A*	2	3	4	2	2	2	2	2	2	5
	*H*_*o*_	0.471	0.176	0.353	0.353	0.412	0.471	0.353	0.235	0.588	0.412
	*H*_*E*_	0.415	0.164	0.424	0.415	0.389	0.498	0.291	0.291	0.457	0.356
	*F*_*IS*_	-0.133	-0.074	0.167	0.150	-0.058	0.056	-0.214	0.190	-0.288	-0.155
6	*A*	2	3	3	2	2	2	2	2	3	4
	*H*_*o*_	0.350	0.150	0.300	0.200	0.350	0.400	0.350	0.250	0.700	0.500
	*H*_*E*_	0.489	0.141	0.339	0.255	0.349	0.480	0.349	0.219	0.540	0.529
	*F*_*IS*_	0.284	-0.062	0.114	0.216	-0.004	0.167	-0.004	-0.143	-0.296	0.054
7	*A*	2	3	3	2	2	2	2	2	3	4
	*H*_*o*_	0.350	0.150	0.300	0.200	0.350	0.400	0.350	0.250	0.700	0.500
	*H*_*E*_	0.489	0.141	0.339	0.255	0.349	0.480	0.349	0.219	0.540	0.529
	*F*_*IS*_	0.284	-0.062	0.114	0.216	-0.004	0.167	-0.004	-0.143	-0.296	0.054
8	*A*	2	4	5	2	2	2	3	2	3	5
	*H*_*o*_	0.301	0.301	0.590	0.349	0.422	0.470	0.494	0.108	0.494	0.518
	*H*_*E*_	0.384	0.294	0.503	0.319	0.451	0.497	0.506	0.164	0.511	0.532
	*F*_*IS*_	0.215	-0.024	-0.173	-0.097	0.065	0.055	0.023	0.340	0.034	0.026
9	*A*	2	2	4	2	2	2	2	2	3	3
	*H*_*o*_	0.339	0.390	0.525	0.356	0.475	0.424	0.559	0.051	0.424	0.627
	*H*_*E*_	0.324	0.403	0.505	0.314	0.493	0.479	0.500	0.050	0.514	0.539
	*F*_*IS*_	-0.046	0.032	-0.041	-0.134	0.037	0.116	-0.119	-0.026	0.176	-0.163
10	*A*	2	4	5	2	2	2	3	2	3	4
	*H*_*o*_	0.400	0.200	0.400	0.300	0.600	0.350	0.200	0.200	0.400	0.400
	*H*_*E*_	0.480	0.186	0.505	0.375	0.495	0.439	0.395	0.180	0.499	0.468
	*F*_*IS*_	0.167	-0.074	0.208	0.200	-0.212	0.202	0.494	-0.111	0.198	0.144
11	*A*	2	4	5	2	2	2	3	2	3	6
	*H*_*o*_	0.432	0.108	0.568	0.216	0.541	0.459	0.297	0.297	0.486	0.622
	*H*_*E*_	0.456	0.104	0.581	0.307	0.482	0.407	0.360	0.290	0.481	0.555
	*F*_*IS*_	0.051	-0.039	0.024	0.295	-0.121	-0.130	0.174	-0.026	-0.011	-0.120
12	*A*	2	6	5	2	2	2	3	2	3	6
	*H*_*o*_	0.536	0.232	0.500	0.554	0.375	0.232	0.411	0.268	0.536	0.464
	*H*_*E*_	0.497	0.213	0.464	0.481	0.500	0.205	0.406	0.305	0.526	0.439
	*F*_*IS*_	-0.077	-0.090	-0.078	-0.152	0.250	-0.131	-0.012	0.121	-0.018	-0.057
13	*A*	2	3	5	2	2	2	3	2	3	5
	*H*_*o*_	0.405	0.262	0.524	0.452	0.429	0.190	0.286	0.452	0.429	0.476
	*H*_*E*_	0.477	0.234	0.535	0.350	0.459	0.172	0.338	0.350	0.489	0.458
	*F*_*IS*_	0.152	-0.117	0.021	-0.292	0.067	-0.105	0.154	-0.292	0.123	-0.040
14	*A*	2	5	5	2	2	2	3	2	3	6
	*H*_*o*_	0.511	0.222	0.556	0.422	0.489	0.156	0.400	0.333	0.533	0.600
	*H*_*E*_	0.475	0.206	0.573	0.437	0.429	0.143	0.336	0.278	0.529	0.552
	*F*_*IS*_	-0.075	-0.080	0.030	0.033	-0.141	-0.084	-0.190	-0.200	-0.009	-0.088
15	*A*	2	3	5	2	2	2	2	2	3	5
	*H*_*o*_	0.571	0.393	0.393	0.286	0.643	0.179	0.250	0.357	0.571	0.321
	*H*_*E*_	0.500	0.327	0.596	0.337	0.459	0.163	0.270	0.337	0.548	0.524
	*F*_*IS*_	-0.143	-0.201	0.341	0.152	-0.400	-0.098	0.073	-0.061	-0.043	0.387
16	*A*	2	3	5	2	2	2	3	2	3	4
	*H*_*o*_	0.514	0.257	0.429	0.486	0.543	0.200	0.571	0.457	0.629	0.514
	*H*_*E*_	0.467	0.232	0.487	0.420	0.441	0.180	0.454	0.382	0.551	0.449
	*F*_*IS*_	-0.101	-0.107	0.121	-0.156	-0.230	-0.111	-0.258	-0.197	-0.140	-0.144
17	*A*	2	6	5	2	2	2	3	2	3	4
	*H*_*o*_	0.409	0.364	0.545	0.318	0.432	0.114	0.341	0.386	0.500	0.568
	*H*_*E*_	0.474	0.339	0.549	0.397	0.456	0.146	0.334	0.407	0.468	0.493
	*F*_*IS*_	0.137	-0.073	0.006	0.198	0.054	0.224	-0.022	0.050	-0.069	-0.154
18	*A*	2	4	5	2	2	2	3	2	3	5
	*H*_*o*_	0.457	0.152	0.457	0.304	0.435	0.217	0.326	0.348	0.565	0.543
	*H*_*E*_	0.471	0.182	0.473	0.364	0.440	0.194	0.368	0.315	0.512	0.534
	*F*_*IS*_	0.032	0.165	0.034	0.164	0.011	-0.122	0.115	-0.105	-0.104	-0.018

*A*: The number of alleles; *H*_*0*_: observed heterozygosity; *H*_*E*_: expected heterozygositiy; *F*_*IS*_: inbreeding cooeffcient

### Population differentiation

#### Pairwise *F*_ST_

**Fig 3:**
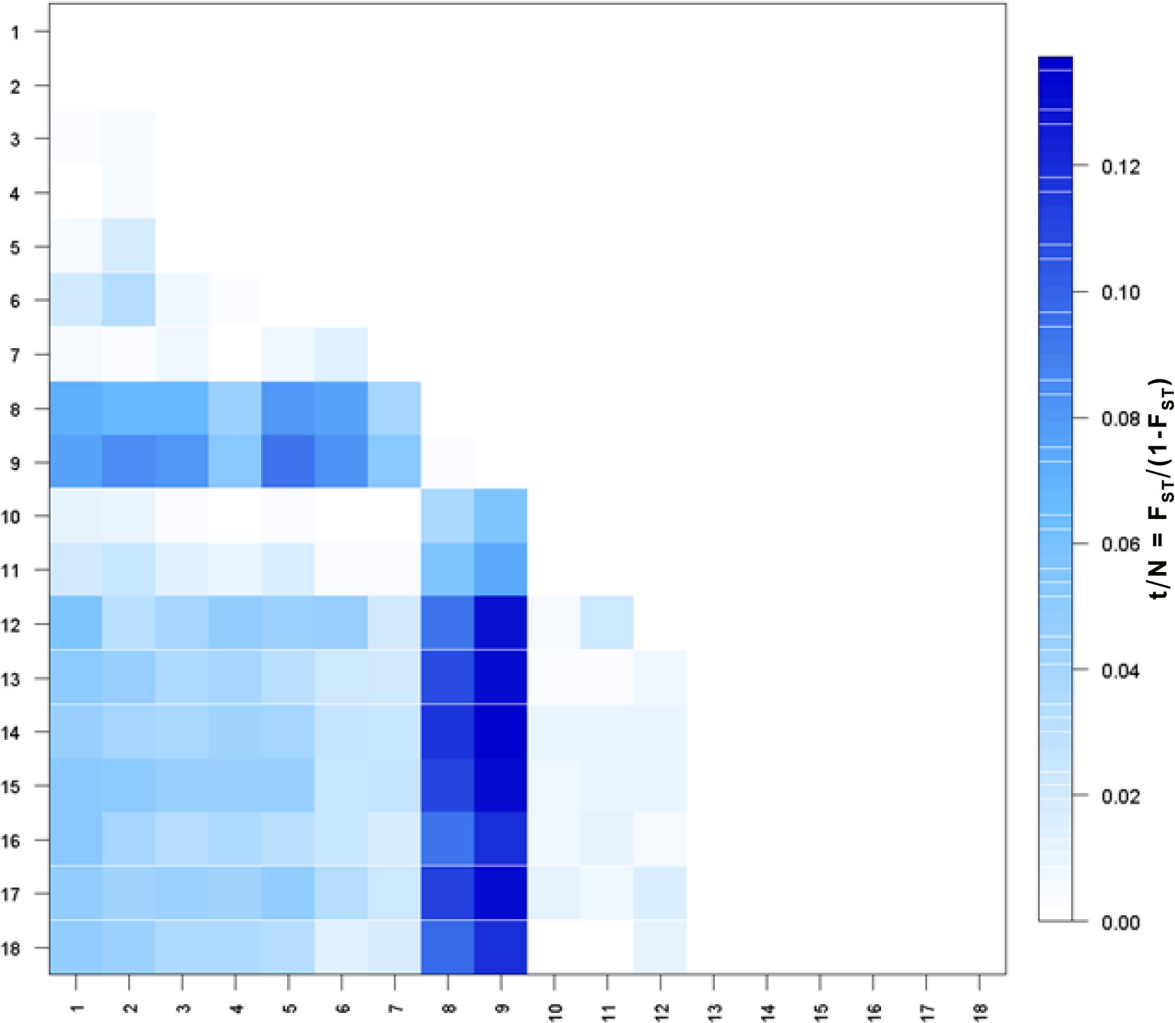
Slatkin’s [53] linearized *F*_ST_ values of 18 localities of Atlantic and Mediterranean swordfish. Population numbers correspond to the sampled localities in Fig 1.

Multilocus pairwise *F*_ST_ values among the 18 populations ranged considerably (<0.001 to 0.121) (Table 3). The comparison between Mediterranean and South Atlantic populations yielded the highest indices of differentiation (*F*_ST_ >0.081 – 0.121). Several sampling localities identified as admixture zones (6–7, 10–11) were excluded when estimating gene flow among North Atlantic (1–5), Mediterranean (8–9) and South Atlantic (12–18) populations. The lowest level of gene flow of about 5 migrants per generation (*Nm*) was estimated between Mediterranean and South Atlantic populations, compared to about 9 migrants per generation between North Atlantic and South Atlantic swordfish (Table 4). Slatkin’s linearized *F*_*ST*_ values (Fig 3) highlights the homogeneity between the Mediterranean sample collected to the east of the Alboran Sea (8) and the adjacent Northeast Atlantic area west of the Strait of Gibraltar (9). The magnitude of these fixation indices also underscore the high degree of differentiation of Mediterranean swordfish with respect to all the Atlantic localities (1–7, 10–18) including those inhabiting the adjacent NE Atlantic waters off both the Iberian Peninsula and the NW African coast. In addition, Fig 2 illustrates that the North Atlantic localities (1–5) are different from the South Atlantic localities (13–18) but also from locality 12 which corresponds geographically to the North Atlantic stock since it is located north of the 5°N management boundary. Linearized *F*_*ST*_ values depict a geographic gradient of differentiation from the Northwest Atlantic to the Southeast Atlantic indicative of mixing between the South Atlantic and North Atlantic populations, primarily in the eastern localities of Iberian, Morocco, and Western Sahara (7, 10–11) and to a lesser extent in the central North Atlantic (6) locality.

**Table 3:** Pairwise *F*_ST_ (lower diagonal) and corresponding significance values (upper diagonal) among the 18 swordfish localities representative of Atlantic and Mediterranean populations characterized in this study (see Table 1).

	1	2	3	4	5	6	7	8	9	10	11	12	13	14	15	16	17	18
**1**		0.401	0.319	0.438	0.272	*	0.161	***	***	0.112	**	***	***	***	***	***	***	***
**2**	0.000		0.108	0.106	*	**	0.190	***	***	0.118	**	**	***	***	***	***	***	***
**3**	0.001	0.006		0.470	0.419	0.226	0.074	***	***	0.393	*	***	***	***	***	**	***	***
**4**	0.000	0.006	0.000		0.429	0.250	0.380	***	***	0.385	0.055	***	***	***	***	**	***	***
**5**	0.003	**0.018**	0.000	0.000		0.411	0.188	***	***	0.410	0.089	**	*	***	**	*	**	*
**6**	**0.018**	**0.033**	0.006	0.004	0.000		0.078	***	***	0.428	0.325	**	*	**	*	*	**	0.080
**7**	0.005	0.003	0.007	0.000	0.007	0.013		***	***	0.423	0.279	***	**	**	**	*	**	**
**8**	**0.066**	**0.063**	**0.064**	**0.042**	**0.074**	**0.070**	**0.038**		0.158	**	***	***	***	***	***	***	***	***
**9**	**0.069**	**0.077**	**0.074**	**0.049**	**0.085**	**0.076**	**0.050**	0.003		***	***	***	***	***	***	***	***	***
**10**	0.009	0.010	0.001	0.001	0.001	0.000	0.000	**0.036**	**0.052**		0.421	0.226	0.318	0.138	0.224	0.179	0.090	0.411
**11**	**0.019**	**0.025**	**0.014**	0.010	0.015	0.003	0.002	**0.054**	**0.069**	0.000		**	0.203	0.084	0.104	0.063	0.103	0.291
**12**	**0.053**	**0.031**	**0.037**	**0.045**	**0.042**	**0.044**	**0.019**	**0.086**	**0.114**	0.005	**0.021**		0.079	*	0.064	0.134	**	*
**13**	**0.047**	**0.045**	**0.035**	**0.039**	**0.031**	**0.023**	**0.020**	**0.097**	**0.117**	0.002	0.004	0.008		0.417	0.443	0.420	0.415	0.412
**14**	**0.044**	**0.040**	**0.036**	**0.040**	**0.039**	**0.026**	**0.023**	**0.105**	**0.121**	0.008	0.009	0.011	0.000		0.382	0.409	0.394	0.421
**15**	**0.049**	**0.049**	**0.043**	**0.044**	**0.043**	**0.023**	**0.025**	**0.100**	**0.117**	0.005	0.009	**0.010**	0.000	0.000		0.301	0.413	0.429
**16**	**0.050**	**0.040**	**0.032**	**0.035**	**0.031**	**0.025**	**0.018**	**0.085**	**0.106**	0.007	0.012	0.005	0.000	0.000	0.002		0.405	0.412
**17**	**0.045**	**0.039**	**0.041**	**0.041**	**0.045**	**0.031**	**0.022**	**0.100**	**0.117**	0.011	0.008	**0.015**	0.000	0.000	0.000	0.000		0.399
**18**	**0.044**	**0.042**	**0.034**	**0.034**	**0.033**	0.014	**0.017**	**0.089**	**0.106**	0.001	0.002	**0.012**	0.000	0.000	0.000	0.000	0.000	

Note: Significant *F*_*st*_ in bold

* = *P* < 0.05

** = *P* < 0.01

*** = *P* < 0.001

**Table 4:** Matrix of migration (Nm) for North Atlantic (NA) (n = 155), Mediterranean (MED) (n = 142) swordfish, and South Atlantic (SA) (n = 256) as calculated in Arlequin v3.5.

	NA	MED
MED	7.2144	
SA	8.5915	4.8516

#### AMOVA

In all alternative hierarchical arrangements tested (two- and three-group; with all localities and excluding admixture areas) with AMOVA (Table 5) the majority of the variation (>90%) in swordfish was contained within individuals. In all arrangements, among-groups (*F*_CT_) differentiation was highly significant (P<0.01). However, the proportions of variation explained by among-groups were slightly larger in two-group AMOVAs, with 6.63% with all localities and 7.05%, excluding admixture areas (localities 6, 10–11), than in three-group arrangements that yielded 5.17% and 6.45% with and without admixture areas, respectively. In addition, the proportion of variance explained by differences among populations within groups (*F*_SC_) was four to ten times larger in the two-groups arrangements than in the three-groups arrangements, with the lowest proportion of variation (0.19%) for the three-groups arrangement without admixture areas (*F*_SC_ = 0.00207, *P*>0.05; North Atlantic: South Atlantic: Mediterranean).

**Table 5:** Hierarchical analysis of molecular variance (AMOVA) of SNP data for 18 samples of swordfish.

	All localities			Excluding admixture areas		
Genetic Structure	Variance component	% of total	Fixation index	Variance component	% of total	Fixation index
	**Two Groups**					
	G1: Atlantic (1–7,10–18)			G1: Atlantic (1–5, 12–18)		
	G2: Mediterranean (8–9)			G2: Mediterranean (8–9)		
Among groups	0.14991	6.63	*F*_*CT*_ = 0.06634**	0.16037	7.05	*F*_*CT*_ = 0.07046**
Among populations within groups	0.03691	1.63	*F*_*SC*_ = 0.01749***	0.04373	1.92	*F*_*SC*_ = 0.02067***
Among individuals within populations	0.03735	1.65	*F*_*IS*_ = 0.01802^ns^	0.02306	1.01	*F*_*IS*_ = 0.01113^ns^
Within individuals	2.03553	90.08	*F*_*IT*_ = 0.09920***	2.04876	90.02	*F*_*IT*_ = 0.09981***
	**Three Groups**					
	G1: North Atlantic (1–7,10–11)			G1: North Atlantic (1–5)		
	G2: Mediterranean (8–9)			G2: Mediterranean (8–9)		
	G3: South Atlantic (12–18)			G3: South Atlantic (12–18)		
Among groups	0.11349	5.17	*F*_*CT*_ = 0.05171***	0.14314	6.45	*F*_*CT*_ = 0.06450***
Among populations within groups	0.00834	0.38	*F*_*SC*_ = 0.00401**	0.00430	0.19	*F*_*SC*_ = 0.00207^ns^
Among individuals within populations	0.03735	1.70	*F*_*IS*_ = 0.05171^ns^	0.02306	1.04	*F*_*IS*_ = 0.1113^ns^
Within individuals	2.03553	92.75	*F*_*IT*_ = 0.07253***	2.04876	92.32	*F*_*IT*_ = 0.07683***

ns = P > 0.05

* = P < 0.05

** = P < 0.01

*** = P < 0.001

Samples (in parentheses) were grouped into alternative groups (G1-G3) according to region of capture, with all localities, or excluding localities identified as areas of admixture (see text for explanation)

#### Principal coordinate analysis

**Fig 4:**
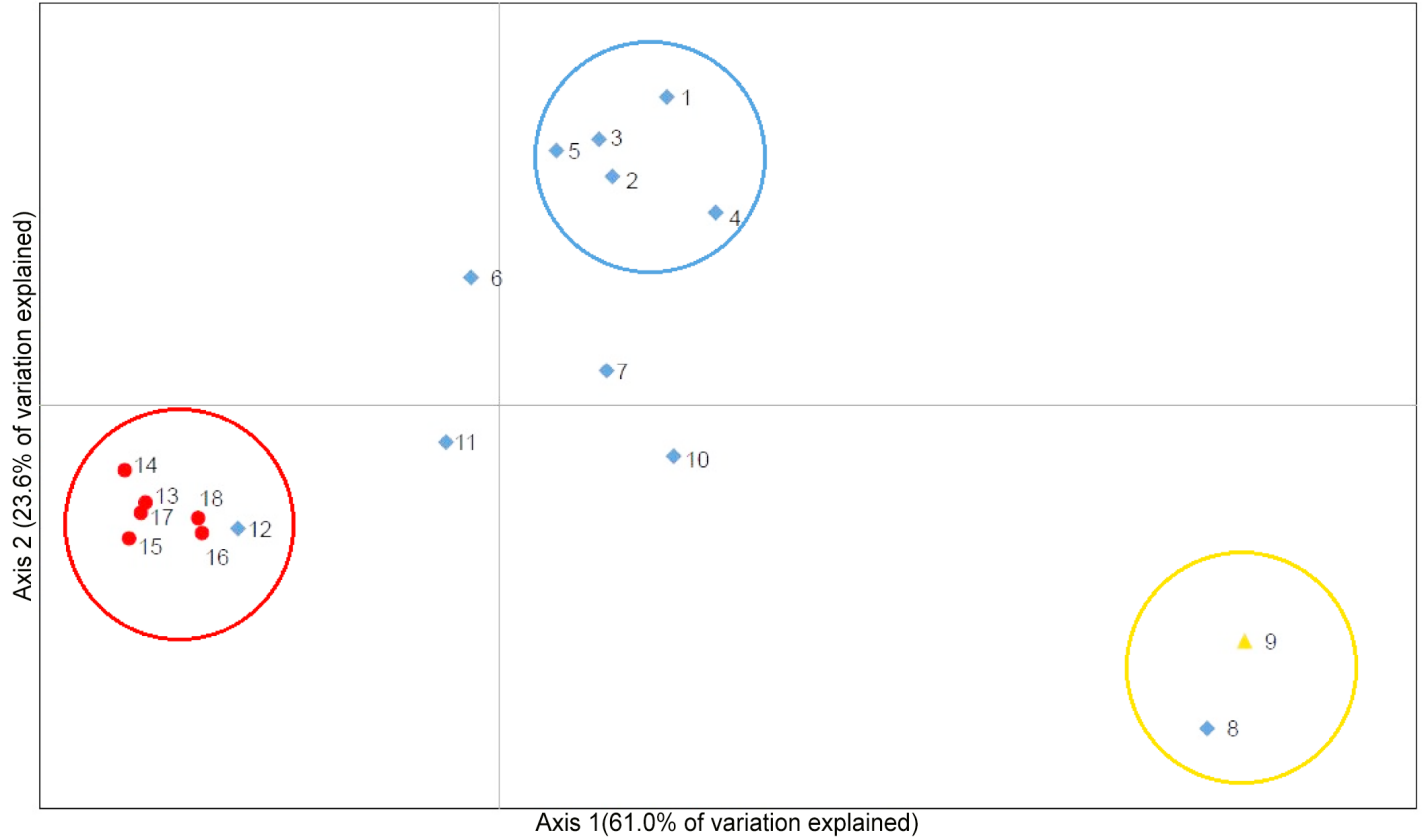
Principal coordinate analysis (PCoA) [54] of 18 localities of Atlantic and Mediterranean swordfish. The numbered population means, corresponding to the sampling localities in Fig 1, are identified by their *current management stock* (yellow triangle = Mediterranean, blue diamond = North Atlantic, red circle = South Atlantic) as defined by the ICCAT management boundaries at 5°N and the Strait of Gibraltar. Colored circles of equal diameter are drawn to display population groupings, red = South Atlantic, blue = North Atlantic, yellow = Mediterranean.

The PCoA of the standardized covariance of pairwise population genetic distances provide concordant evidence of locality assignment to a minimum of three separate swordfish populations (Fig 4). The first three axes accounted for ~93.86% of the variation among populations with Eigen values of 0.847 and 0.328 for the first and second axes, respectively. Based on the clustering patterns, three groups can be identified corresponding to North Atlantic, South Atlantic, and Mediterranean populations. Loadings in the first axis contrast the Mediterranean from the South Atlantic, whereas the second axis contrasts the Mediterranean with the North Atlantic. The North Atlantic cluster, is not as tightly grouped as other populations, and is comprised of the Gulf of Mexico (1), Southeast U.S. Coast (2), Northeast U.S. Coast (3), Lesser Antilles (4), and east of Flemish Cap (5) samples. The sample collected west of the Strait of Gibraltar (8), which for management purposes corresponds to the North Atlantic stock, cluster tightly with the Mediterranean sample (9), and together they form the Mediterranean group. Finally, the South Atlantic group included the samples from Cape Verde (12), collected north of the current boundary between northern and southern Atlantic swordfish stocks, which cluster tightly with the samples from Equatorial Brazil (13), Gulf of Guinea (14), Central South Atlantic (15), Brazil (16), Brazil-Uruguay (17), and Namibia (18). The remaining samples occupy an intermediate position, with central North Atlantic (6) and Iberian (7) samples associating more closely with the North Atlantic group, whereas Morocco (10) and Western Sahara (11) samples more closely with the South Atlantic group.

### Bayesian genetic clustering analyses

#### STRUCTURE analysis

Evaluation of the mean posterior probabilities from multiple STRUCTURE analyses (*K* = 1–10) revealed that the mean ln *P*(D) increased from *K* = 1–3 and then sharply decreased when *K* ≥ 4 (see supporting S1 Fig) indicating that *K* = 3 reflects the majority of the genetic structure in the dataset [55]. Estimated Δ*K*’s revealed two high Δ*K* peaks of similar magnitude corresponding to *K* = 2 (Δ*K*_2_ = 35.887) and *K* = 3 (Δ*K*_3_ = 33.6548), followed by very low Δ*K* (< 5.0) values (see supporting S1 Fig). Mediterranean localities (8–9) were removed from the analysis to test whether the strong differentiation of the Mediterranean from the Atlantic was causing an underestimation of *K*. The evaluation including only North Atlantic and South Atlantic localities identified a single Δ*K* peak at *K* = 2 (not shown) congruent with the genetic heterogeneity between the North and South Atlantic populations of swordfish. While Evanno’s Δ*K* method seeks to detect the uppermost hierarchical level of population structure the method is less reliable at lower levels of genetic differentiation and may underestimate *K* [64]. Therefore, the estimation of *K* using the *ad hoc* evaluation of mean posterior probabilities from multiple analyses of *K* [55] appears to be more appropriate in this instance than the Δ*K* approach [60].

**Fig 5:**
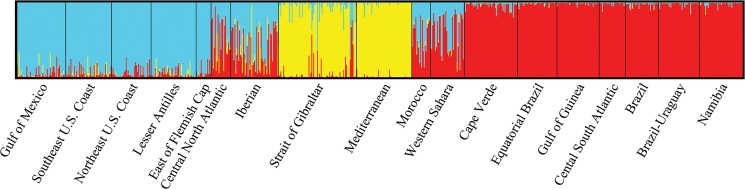
Bayesian individual assignment of Atlantic and Mediterranean swordfish in STRUCTURE v2.3 [55] using no admixture, correlated alleles, and LOCPRIOR models and inferred from 50 independent runs of *K* = 3 using 100,000 MCMC iterations and a burn-in period of 100,000. Estimated individual membership coefficients ($\hat{Q}$) are sorted by sampling locality and correspond to the numbered localities in Fig 1.

Average population posterior probability memberships ($\bar{Q}$) of the 18 localities (see supporting S4 Table) and individual assignments at *K* = 3 were concordant with results from PCoA, AMOVA, and *F*_*ST*_ analyses (Fig 5). Localities 1–5 had average values of ancestry probabilities ($\bar{Q}$) belonging to the North Atlantic > 0.90 with no individuals of South Atlantic or Mediterranean origin. Swordfish collected in the central North Atlantic (6) clustered between the North Atlantic and the South Atlantic indicating a potential mixing area, however sample size was small (n = 20). The Iberian (7) sample clustered slightly closer with the North Atlantic ($\bar{\text{Q}}$= 0.55) and represented an area of mixing with at least one individual belonging to the Mediterranean with the rest either to the North Atlantic or the South Atlantic. No North Atlantic or South Atlantic swordfish were identified within the Mediterranean sample (9) and the area west of the Strait of Gibraltar (8) clustered ($\bar{\text{Q}}$= 0.85) with the Mediterranean (9). In fact, 77 out 83 individuals sampled (93%) immediately to the west of the Strait of Gibraltar (8) were identified by STRUCTURE as Mediterranean. Of the remaining individuals, three swordfish (3.6%) were identified as North Atlantic and another three (3.6%) as South Atlantic. Four Mediterranean swordfish were also identified in the Iberian sample (7) as follows: three at 37.5°N 12.5°W and one at 37.5°N 22.5°W, accounting for roughly 11% and 5% of the total number of individuals sampled respectively in these two locations.

The area off the Atlantic coast of Morocco (10) was also identified as an area of mixing with individuals of North Atlantic and South Atlantic ($\bar{\text{Q}}$= 0.52) origin. Although the sample of Western Sahara (11) contained a majority of individuals assigned to the South Atlantic ($\bar{\text{Q}}$= 0.57), it is a zone of admixture containing also individuals of North Atlantic origin. Swordfish from Cape Verde (12) along with all the individuals sampled south of 5°N (13–18) were assigned to the South Atlantic ($\bar{\text{Q}}$> 0.95), with no North Atlantic or Mediterranean migrants. STRUCTURE results using only *CaM* revealed that while this locus drives the heterogeneity between South Atlantic and North Atlantic populations, alone it fails to differentiate between North Atlantic and Mediterranean populations. Conversely, Bayesian analysis using all the loci except *CaM* differentiated between North Atlantic and Mediterranean populations. Further, this analysis also discriminates North Atlantic from South Atlantic swordfish, although posterior probabilities were higher when *CaM* was included (see supporting S2 Fig).

#### GENELAND analysis

**Fig 6:**
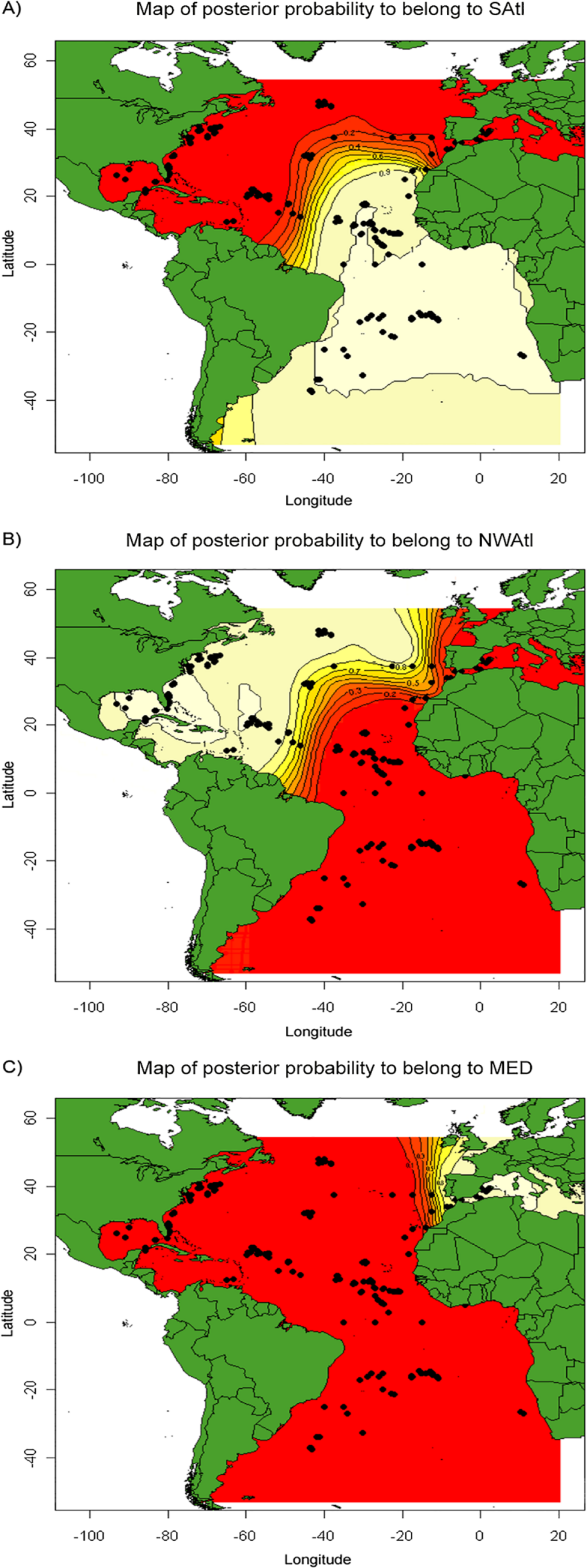
Posterior probability contour maps of Atlantic and Mediterranean swordfish calculated in GENELAND [62] with an uncertainty on coordinate value = 30°, correlated allele frequency and spatial models, and inferred from 20 independent runs of *K* = 3 using 100,000 MCMC iterations and a thinning of 100. Posterior probability contours range from 1.0 in light yellow to 0.1 in red for membership to the (A) South Atlantic population vs. all other populations, (B) North Atlantic vs. all other populations, and (C) Mediterranean vs. all other populations. Black circles correspond to sampling locations and may indicate more than one individual. Posterior probability contours in areas of limited and/or no sample coverage are extrapolations and may therefore contain error (e.g. no northern Portugal sampling extends the Mediterranean posterior probability of membership).

The *K* value with the greatest density on the MCMC chain after a burn-in of 10,000 was *K* = 4. However, inspection of map of posterior probabilities identified one cluster as a ‘ghost’ with no associated samples (not shown). Further, this ghost cluster was unstable, as it’s placing or presence differed among replicate runs. The existence of ‘ghost’ clusters has been reported as an artifact of GENELAND which tends to overestimate *K* in a comparison to other Bayesian genetic clustering algorithms [65]. Accordingly, GENELAND was run with the optimal value (*K* = 3) identified by STRUCTURE. Localities 1–5 and part of 7 comprised the North Atlantic ($\bar{\text{Q}}$> 0.90), localities 8 and 9 the Mediterranean ($\bar{\text{Q}}$> 0.90), and localities 12–18 the South Atlantic ($\bar{\text{Q}}$> 0.90). The individual posterior probabilities assigned in GENELAND (Fig 6) identified the region corresponding to the central North Atlantic (6) and Western Sahara (11) samples as areas of mixing between the North Atlantic and South Atlantic populations. The samples corresponding to the North Atlantic area west of the Strait of Gibraltar (8) have the genetic signature of the western Mediterranean (9), whereas swordfish from the Atlantic locality of Morocco (10) were identified as either Mediterranean or North Atlantic. The Iberian sample (7) is comprised of three subsamples located at 37.5°N 22.5°W (n = 19), 37.5°N 17.5°W (n = 5), and 37.55°N 12.5°W (n = 27), with a North Atlantic origin assigned by GENELAND of > 80%, >70%, and <50%, respectively. Since the precise geographic coordinates of capture for the individuals in these Atlantic subsamples were recorded only with a precision of 2.5° latitude by 2.5° longitude, and because the subsample sizes are small, these estimates are imprecise.

## Discussion

### Nuclear loci evaluation

Genetic population structure studies using nuclear DNA have traditionally excluded adaptive markers to avoid introducing bias to estimates of migration rates and effective population sizes [66]. In this study we attempted to identify variation in non-coding nuclear introns to maximize the potential of characterizing neutral loci. Of the 26 SNPs assayed 25 were contained in the introns of 10 nuclear genes, with the exception of a SNP corresponding to a third codon synonymous substitution in one exon of the ATPsβ gene. Although introns are generally assumed to be neutral, some may contain transcription regulation or splicing control elements that alter gene expression [67]. Introns may also experience purifying and/or positive selection [68] or the effects of hitch-hiking (i.e. linkage between a non-coding variant and a gene under selection) as reported in Atlantic cod [69]. Therefore deviations from HWE, linkage disequilibrium tests, and outlier analysis are commonly applied to detect potentially adaptive loci. In our study exact HWE [42] and linkage equilibrium [43] tests were not significant after Bonferroni corrections. Similarly the the *F*_ST_ outlier analysis failed to identify any loci characterized as subject to selection. Given that the number of loci analyzed in the current study is small (n = 10), the associated error in the calculation of global neutral *F*_*ST*_ could be large. Accordingly, future studies utilizing genomic technologies will certainly yield greater numbers of loci to conduct more conclusive outlier tests.

Alternatively, outlier and non-neutral SNPs can provide insight into adaptive genetic diversity within and amoung populations [70, 71] which may be useful for resolving fine-scale population connectivity, admixture, mixed stock analysis, and identifying local adaptation for conservation or resource management [72–74]. Studies utilizing adaptive loci in Atlantic cod [75], Atlantic herring [74, 76], Atlantic salmon [77], and Pacific lamprey [78] reported that fewer loci and smaller sample sizes were required to detect population differentiation in marine fishes when using outlier loci as compared to neutral loci. Muths, Le Couls (79) hypothesized that the inclusion of adaptive loci in future genetic analses of large pelagic fishes, specifically swordfish, may identify additional evolutionary significant population units than analyses relying on only neutral loci. However, since the inclusion of outlier loci may violate model assumptions used for individual assignment, it is important that outlier loci included in such analyses are carefully evaluated and presented with comparative results using neutral loci [74]. While the ranking analysis identified *CaM* as the most informative locus for distinguishing North and South Atlantic swordfish populations, this locus alone fails to differentiate between North Atlantic and Mediterranean swordfish populations. Further, STRUCTURE results without *CaM* (see supporting S2 Fig) show that the remaining loci were capable to discriminate between North and South Atlantic swordfish. Accordingly, the high resolution of population differentiation reported in this study among North Atlantic, South Atlantic and Mediterranean swordfish cannot be attributed to *CaM* alone. In fact, we demonstrate that the inclusion of the additional loci increases the power of population discrimination.

### Atlantic swordfish population differentiation

Analyses of multilocus nuclear SNP data indicate that Atlantic swordfish can be subdivided into at least three populations, namely Mediterranean, North Atlantic, and South Atlantic. The presence of these populations is supported by linearized pairwise *F*_ST_ [52, 53], AMOVA [50], PCoA [54], and Bayesian genetic clustering using both in STRUCTURE [55, 57, 59] and GENELAND [62]. Though no geographic barriers prevent gene flow among North Atlantic, South Atlantic and Mediterranean swordfish populations, the number of migrants among populations is small (Table 4). Additionally, the observed temporal stability in allele frequencies within the Northwest Atlantic, South Atlantic, and Mediterranean populations also suggests limited gene flow among these swordfish populations. This temporal stability is perhaps best evident in the parity of genetic signature between the larval Gulf of Mexico samples and the samples of adult Northwest Atlantic swordfish collected over a decade prior. Based on these results the North Atlantic population extends from the tropical spawning areas in northwestern Atlantic waters [80, 81] to 50°W. In the feeding areas north of 40°N the range of the North Atlantic population extends to the Azores and may extend at that latitude as far east as 15°W. The South Atlantic population extends from 50°S through the South Atlantic tropical and equatorial spawning areas [17] to 20°N at 40°W and 25°N along the African coast.

The Mediterranean population extends past the Strait of Gibraltar, possibly as far as 8°W, with little evidence of Atlantic swordfish entering the Mediterranean Sea. These results are concordant with the phylogeographical association of mtDNA lineages previously reported [7, 13, 17, 56] and also with evidence derived from fisheries data [82]. The Mediterranean population is identified here as the most divergent of the three populations, and these findings agree with other studies using mtDNA, scnDNA (see [25] for summary), and microsatellite loci [29, 30].

The Bayesian clustering analyses based on multilocus SNPs presented here are not consistent with analyses of global swordfish population structure using microsatellites [28, 30]. Those studies reported extremely low levels of differentiation between North and South Atlantic populations (*F*_ST_ ≈ 0.0015), precluding individual cluster assignment. By contrast, Kasapidis et al. [29] argued that the considerably higher estimates of differentiation reported with nuclear SNPs, and specifically with *CaM*, is strong adaptive selection. Such explanation, however, fails to explain the concordance between mtDNA and nuclear SNPs data (reviewed in [25]), including the current study. Instead, such agreement suggests that the differentiation between North Atlantic and South Atlantic swordfish is the consequence of population subdivision and low levels of gene flow.

The shallow levels of differentiation between North and South Atlantic using microsatellites could be due to allele size homoplasy in combination with large effective sizes [83] of swordfish populations and the high mutation rates characteristic of these type of loci [84]. Alternatively, the failure to find differences using microsatellite analyses within the Atlantic [28–30] could be due to the small number of loci (4–10) characterized in those studies, or because the samples employed to represent the North Atlantic came from areas identified here as admixture zones within the Northeast Atlantic. Accordingly, a stronger signal of differentiation between South Atlantic and North Atlantic swordfish may be obtained by increasing the number of microsatellite loci aimed to characterize representative samples from reproductive areas in the Northwest Atlantic. Yet, in other highly migratory marine fishes the strong differentiation revealed with mtDNA has not been obtained with microsatellites, and such discordance was interpreted as evidence of male mediated gene flow in bigeye tuna (*Thunnus obesus*)[16], bluefin tuna (*T*. *thynnus*) and yellowfin tuna *(T*. *albacares*) [85], sandbar shark (*Carcharhinus plumbeus*) [86], and shortfin mako (*Isurus oxyrinchus*) [15], without considering alternative hypotheses. In blue marlin (*Makaira nigricans*) mitochondrial cytochrome b gene sequences [87] and RFLP data of the mtDNA molecule [88] revealed a pronounced signal of inter-oceanic differentiation that was considerably higher compared to allozyme and scnDNA data [89]. Such disparity was attributed to the enhanced effect of genetic drift under migration-drift equilibrium resulting from the fourfold lower effective population size of mitochondrial than nuclear molecules, and while the effect of male biased inter-ocean exchange could not be rejected, it was not required to account for the observed disparity. Computer simulations and power analyses conducted later on the blue marlin allozyme and scnDNA data together with newly generated microsatellite and whole molecule mtDNA RFLP data [90], indicated that the large range of *F*_ST_ values among marker classes and among loci within a marker class were consistent with the same population level *N*_*e*_ *m*, given differences in both mode of inheritance and genomic sampling variance among estimates. Further, one microsatellite locus (Mn8) yielded a pattern concordant with mtDNA regarding both the strong inter-oceanic differentiation, and inferred unidirectional gene flow of Indo-Pacific blue marlin into the South Atlantic. In swordfish, the similarity in levels of differentiation between North and South Atlantic populations using mtDNA (*F*_ST_ = 0.0244; [17]) and multilocus nuclear SNPs (*F*_ST_ = 0.0393; this study), is not consistent with gender-biased gene flow. Considering that both swordfish and blue marlin females attain larger sizes than males and are more abundant at higher latitudes [81, 91, 92] where inter-oceanic gene flow would be facilitated (e.g., around southern Africa), it would be highly unlikely that male biased dispersal explains the observed inter-oceanic allele frequency discrepancies among nuclear and mitochondrial markers in both species. Further, in the Atlantic the differences between the sex ratios at-size seem to support the hypothesis that mature swordfish females are more mobile, tending to cover (in terms of probability) broader geographic areas to sustain the energetic requirements required to support the high production of eggs, whereas the migration of males would be generally more restricted and conditioned by the lower average body biomass and the lower energy requirements in comparison to the females to produce spawning products [92]. Thus in spite of the higher mobility and gene flow potential of females no gender-biased gene flow was detected in Atlantic swordfish.

### Atlantic swordfish population subdivision and admixture

Previous studies of Atlantic swordfish population structure only broadly identified potential areas of admixture between the North Atlantic and Mediterranean populations [7, 31] and between the North Atlantic and South Atlantic populations [17, 18, 26] and offered no estimates of the contribution of each population. The Strait of Gibraltar is the current management boundary utilized by ICCAT to separate the Mediterranean and the North Atlantic populations. Multilocus analyses of SNPs with STRUCTURE (Fig 5) and GENELAND (Fig 6C) suggests that the Mediterranean stock extends past the management boundary to around 10°W into North Atlantic waters. However, given that the mapping algorithm of GENELAND extrapolates posterior probabilities into areas of limited or no sample coverage some of the posterior probability curves should be viewed with caution. Specifically, since no samples off the northern coasts of Portugal or Spain were characterized, the posterior probability curves in this region should not be interpreted as evidence of the Mediterranean population extending around the Iberian Peninsula into the Bay of Biscay and the Celtic Sea (Fig 6C). Taken all together, Mediterranean swordfish exiting through the Strait of Gibraltar appear to remain primarily in the shallower Atlantic shelf waters off the northwest African and Iberian coasts, with occasional migrants venturing farther into North Atlantic waters, perhaps as far as the Azores [30, this study]. Movements of mature swordfish inhabiting the shelf area west of the Strait of Gibraltar into the Mediterranean, putatively associated with spawning behavior around the Balearic Islands, occur from May to the beginning of July [82]. Later in the year, between August-November a migration associated with feeding behavior in the opposite direction (east to west) across the Strait takes place with no reproductively active swordfish and with 15% of the catch corresponding to juveniles [82]. Given that our samples are limited to summer and fall when migrations are believed to take place, the expected reduction in frequency of Mediterranean swordfish in this Atlantic region during the winter and spring cannot be confirmed at this time. In a previous study using mtDNA CR-I sequence data, Alvarado Bremer et al. [93] documented no Atlantic swordfish east of the Strait of Gibraltar. Similarly, in this study, no Atlantic swordfish were identified within the Mediterranean Sea (n = 59). It should be noted that in this study Mediterranean swordfish were only sampled in the western Mediterranean, specifically in the area east of the Alboran Sea and south of the Balearic Islands, where the presence of swordfish juveniles has been documented [94] and reproductive behavior is expected to occur [82]. If North Atlantic swordfish were to enter the Mediterranean, their presence would be best documented in this region. Further, the low frequency of movements of Atlantic swordfish into the Mediterranean is also supported by the Bayesian analysis of microsatellite data from 602 western and eastern Mediterranean swordfish in which only three individuals of putative Atlantic origin were identified [30]. Altogether it appears that oceanographic features and or behavioral cues limit the extent of admixture of Atlantic and Mediterranean swordfish populations, with the majority of the contact confined to the shallower waters of both the northwest African shelf and the Gulf of Cadiz.

While the Strait of Gibraltar and associated oceanographic and geophysical features may constrain the admixture between North Atlantic and Mediterranean populations, no such physical barrier separates the populations of North Atlantic and South Atlantic swordfish with admixture occurring over a considerably broader geographic area. A proposal to move the current management boundary separating the North Atlantic from the South Atlantic populations from 5°N to about 15°N was suggested by Chow et al. [26] on the basis of significant differences in allele frequency of *CaM*. Our analyses with PCoA (Fig 4), STRUCTURE (Fig 5), and GENELAND (Fig 6) are in agreement with the extension of the South Atlantic swordfish stock past the current 5°N boundary but suggest an extension even farther north, past Cape Verde (12) to around 20°N. This boundary does not conforms to a horizontal line extending from the Caribbean Sea to the African coast. Instead, the 0.90 isoline of GENELAND is more akin to a step extending from about 0°N 45°W north to 25°N 45°W, and from there almost horizontally towards the African coast (Fig 6). To the north and west of this boundary, an admixture zone begins around Western Sahara (11) and continues north to Morocco (10) and the Iberian sea (7) extending west towards the central North Atlantic (6) and then south towards the northern coast of Brazil. An interesting feature of the pattern of admixture between northern and southern stocks is that the relative proportion contribution to neighboring populations is asymmetrical, with South Atlantic swordfish moving past this boundary towards the north, but with no swordfish of North Atlantic origin moving into the range of the South Atlantic population, reminiscent of the asymmetric contact between the North Atlantic and Mediterranean swordfish populations.

Posterior probability membership contours generated by GENELAND (Fig 6) could be used for mixed stock allocation in the identified areas of stock admixture, with the caveat that the sensitivity of spatial Bayesian clustering, particularly when identifying linear boundaries, is contingent upon sampling coverage [95] as well as initial model assumptions. However, altering initial model assumptions did not alter the general pattern of genetic differentiation reported (not shown). Seasonal displacements of North and South Atlantic swordfish, as well as sampling in regions not properly represented here (e.g., 20–25°N and 30–40°W), may change the extent and geographical placement of admixture, but are not expected to alter the general pattern of differentiation separating these two populations (see notes below regarding reduced abundance in these areas derived from catches).

### Potential barriers to gene flow

In the absence of a geographic barrier to explain the asymmetric pattern of admixture separating north and south populations of Atlantic swordfish, biological, behavioral, and oceanographic impediments to gene flow need to be examined. Conventional tag-recapture data [20, 96], though biased in its coverage to primarily the Northwest Atlantic fishery, indicates that swordfish tagged west of the Azores generally follow similar patterns of directional migration, west by southwest, back to the primary breeding grounds of the North Atlantic indicative of spawning and feeding ground fidelity, whereas swordfish tagged east of the Azores (of which there are far fewer records) tend to move east by southeast. Interestingly, of the few swordfish tagged between 15°N and 30°N, movement generally leads east by northeast. While conventional tagging data may mirror some of the same molecular distribution trends inferred in this study, these data are heavily biased to areas of high fishing effort [20] and the reporting tag-recapture rates among fleets. Although limited by their relative short duration, Pop-up satellite archival tag (PSAT) data has yielded similar evidence of seasonal spawning and feeding ground migrations in the Northwest Atlantic [22, 23].

Recently, Abascal et al. [24] reported the PSAT tracks for 10 Atlantic swordfish tagged in the North Atlantic as part of a tagging study aimed to characterize diel vertical diel behaviors and horizontal seasonal migrations and habitat preferences of this species. Seven tracks corresponded to swordfish tagged around 35°N-45°N in latitude and 40°-50°W in longitude. The other three tracks were of swordfish tagged in a southeastern area of the North Atlantic to the north of Cape Verde archipelago, around 20°N-23°N in latitude and 20°W-25°W in longitude. The movements of these 10 swordfish are concordant with the results obtained with STRUCTURE, with four tracks indicating movements within areas (two tracks each) identified here as North Atlantic (localities 3 and 5) or South Atlantic (locality 12) populations, whereas six swordfish moved in and out of sampling areas where the posterior probabilities suggest admixture zones (i.e., locality 6, for the fish tagged in the northern area, and 6, 7 and 11, for fish tagged in the southern area). However, one PSAT deployed in the northern area after spending several months in the central North Atlantic admixture zone, spent the last six months of a year at liberty within locality 12 (Cape Verde), identified here as an area belonging to the South Atlantic population. Altogether, only one of 10 swordfish tracked were in conflict with the probability assignments (>0.95) of STRUCTURE. By contrast, concordance of the PSAT tracks with GENELAND’s North-South Atlantic population boundaries (0.10–0.90 confidence limit) occurred in seven out of ten swordfish. Two swordfish tagged in the northern area, crossed the 0.90 isoline of assignment to the South Atlantic population. Conversely, one of three tracks of swordfish tagged in the southern area crossed the 0.90 confidence isoline delimiting the North Atlantic population. However, none of the swordfish tagged either in the northern or southern areas of the North Atlantic crossed into the respective 1.0 probability isolines delimiting the North Atlantic and South Atlantic swordfish populations, respectively.

### Within-ocean differences in patterns of genetic differentiation

Strong natal fidelity, with admixture primarily confined to feeding grounds, has been proposed as an explanation for the unparalleled intra-oceanic differentiation separating North Atlantic and South Atlantic swordfish populations [17]. By contrast, within the Pacific Ocean [97] and along the Mediterranean Sea [31] differentiation of mtDNA appears to conform to isolation by distance (IBD), although such a pattern in the Pacific Ocean is not supported by the multi-locus analysis of nDNA SNP data [98]. Conversely, in the Indian Ocean the presence of at least two potential genetic populations was suggested [18, 99, 100], but this interpretation was rejected recently [79] in favor of panmixia since the frequency distribution of mitochondrial and microsatellite markers using a more ample sampling coverage of that basin including multiple seasons was homogeneous. Further Muths et al. [79] propose that the presence of distinct swordfish populations respectively within the Atlantic and Pacific Oceans could be partly explained by the existence in those basins of two separate hemispheric oceanographic systems with corresponding discrete feeding areas for the respective populations with limited or no admixture. The association of genetic differentiation with the existence of separate feeding areas as proposed by Muths et al. [79] contrasts dramatically with the interpretation of spawning site fidelity as the driving force behind the observed genetic heterogeneity of swordfish populations. Accordingly, maximum levels of genetic differentiation using both mtDNA and nuclear SNP data were obtained when separate breeding areas were compared [17]. In the current study restricting the analysis to spawning areas yielded higher *F*_ST_ values for all loci while at the same time increased the *H*_e_ values for several loci (e.g. Act2α, *CaM*, Mlc2). This result supports the hypothesis that samples from feeding grounds in both the NW Atlantic and the South Atlantic Ocean (i.e., Gulf of Guinea) may be more prone to population admixture as suggested by Alvarado Bremer et al. [17]. Similarly, spawning site fidelity is also supported by the evidence of the admixture of North Atlantic and Mediterranean swordfish populations in the feeding area to the west of the Strait of Gibraltar by both mtDNA [25] and nDNA data (this study). The concordance of mtDNA and nDNA data was also reported for the separation of South Atlantic from Indian Ocean swordfish [79]. Such concordance of nuclear and mtDNA data in the separation of swordfish populations inhabiting the Mediterranean, North Atlantic, South Atlantic and Indian Ocean basins, is particularly relevant given reported differences in rates of dispersal of swordfish males and females.

While fidelity to spawning and feeding grounds may explain low levels of gene flow between North and South Atlantic swordfish, it doesn’t fully explain why admixture only seems to occur in the Northeast Atlantic waters east of about 50°W and south of 30°N. Chow et al. [26] suggested that changes in oceanographic parameters (e.g. salinity, dissolved oxygen, temperature, etc.) in deep waters might explain the strong genetic break that their *CaM* data revealed since swordfish are deep divers. Nutrient upwelling, caused by prevailing winds off the western coast of Africa, creates a large hypoxic zone in the eastern tropical Atlantic [101] that apparently correlates with the inferred area of population differentiation identified in this study (see supporting S3 Fig). Sensitivity to oceanographic conditions in deep waters has been documented in other billfish [102] and swordfish are known to dive substantially deeper than other billfish [21], and thus may be more sensitive to changes in concentrations in DO at depth, which might be used as a cue by North Atlantic swordfish not to move south to the areas occupied by South Atlantic swordfish.

**Fig 7:**
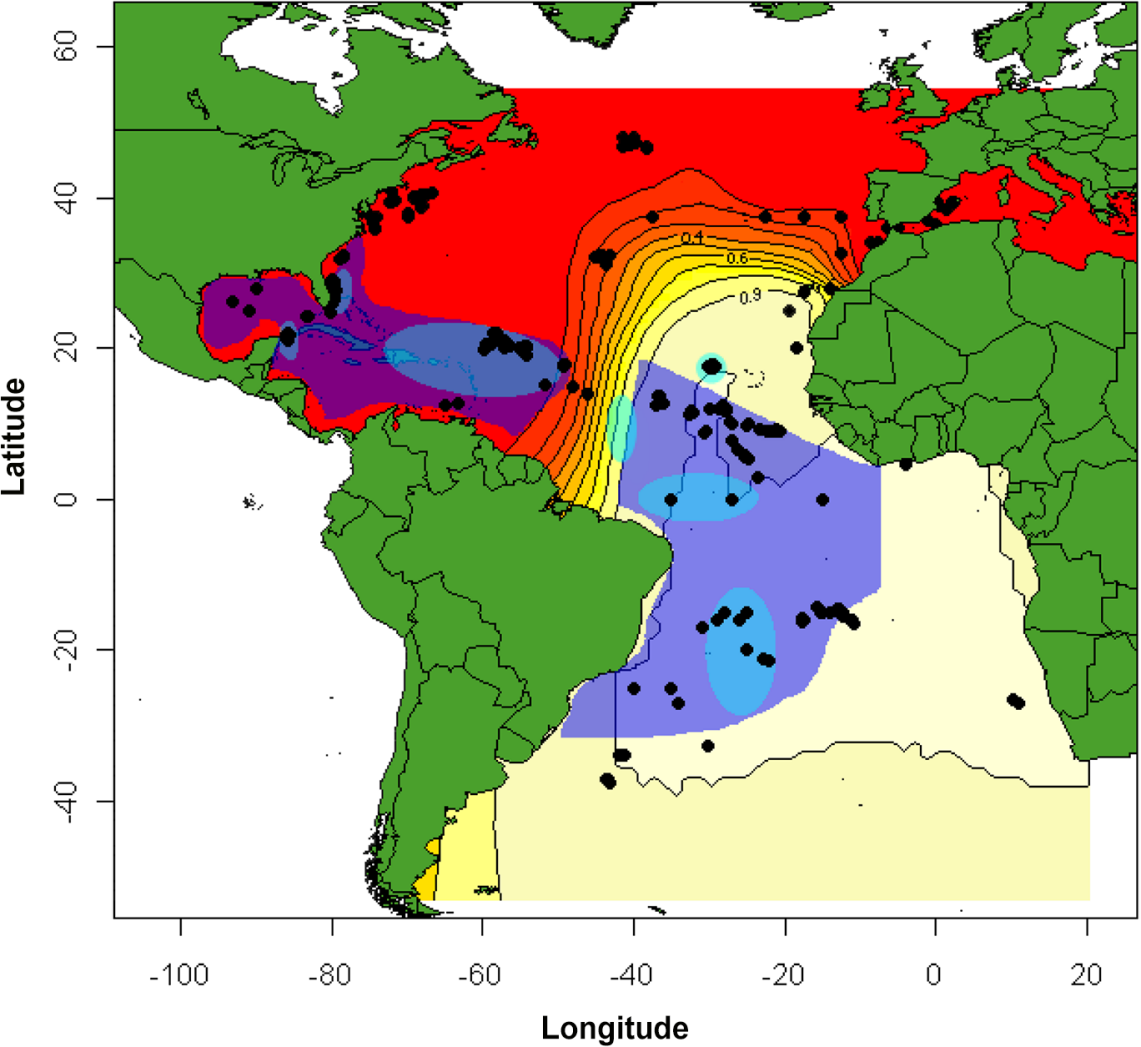
GENELAND map of posterior probability of membership to the South Atlantic population overlaid the regions of reproduction of Atlantic swordfish summarized by Alvarado Bremer et al. [17]. Shaded in dark blue are the Northwest Atlantic and South Atlantic areas where eggs and or early life history stages have been collected. The areas shaded in lighter blue correspond to areas where females with high-gonadal indices and/or hydrated oocytes have been collected that coincide to areas of biased male to female sex-ratios [104].

Other oceanographic features are known to acts as barriers to dispersal in proximity to coastal areas. For instance while recognizing that the Amazon discharge would not be expected to affect the movements of adult highly migratory pelagic fish, Chow et al. [26] hypothesized it may act as a freshwater barrier to passive larval dispersal between the Caribbean and Brazilian provinces. This feature geographically coincides with the posterior probability break between North Atlantic and South Atlantic populations identified by STRUCTURE and GENELAND, as well as an inferred reported break in potential spawning areas [17] (Fig 7). Further, the same region coincides with areas where historical catches of adult swordfish are low, and if catches properly reflect abundance (Fig 6), then the low density of adults in this region together with the Amazon discharge preventing the admixture of larvae from northern and southern breeding areas, as hypothesized by Chow et al. [26] could together promote the observed patterns of genetic differentiation between northern and southern populations of swordfish. Finally, the population structure of Atlantic swordfish may be influenced by sea surface currents as reported in other pelagic species (e.g. blue marlin [103]). The North Atlantic gyre, the Canary current, and the Atlantic equatorial counter current all associate with the population differentiation of North Atlantic and South Atlantic swordfish, and may explain why population admixture is primarily confined to Northeast Atlantic waters.

## Conclusions and Future Studies

The findings of this study can be summarized as follows: first, significant differentiation among Mediterranean, North Atlantic, and South Atlantic swordfish populations is confirmed with very low rates of gene flow, particularly when compared to other highly migratory fishes. Second, population admixture between the North Atlantic and the South Atlantic population, and to a much lesser extent with the Mediterranean, is primarily confined to the Northeast Atlantic and in both cases appears to be unidirectional, from the Mediterranean to the NE Atlantic and from the South Atlantic to the NE Atlantic. Third, the genetic partitioning of these populations differs from currently recognized management boundaries. Fourth, and lastly, while the oceanographic and/or behavioral barriers to gene flow are not yet identified, future tagging, genetic, oceanographic, or physiological studies should focus efforts to areas of admixture in the Northeast Atlantic and areas where individuals were assigned with high posterior probabilities, i.e. North and South Atlantic spawning and feeding grounds. Additionally, future genomic studies utilizing large numbers of SNPs may provide insight into whether adaptive genetic variation is responsible for the fine-scale population structure here reported.

## Supporting Information

S1 Fig Bayesian cluster (K) estimation. The estimation of the number of clusters (K) in the STRUCTURE v2.3 [55] analysis of Atlantic and Mediterranean swordfish using (A) the ad hoc approach of Prichard et al. [55] and the mean posterior probability of the data (L(K)) and (B) ΔK approach of Evanno et al. [60]. Twenty independent runs for each K value (1–10) were performed using 100,000 MCMC iterations with a burn in period of 100,000.
(TIF)

S2 Fig Relative importance of CaM in STRUCTURE analysis. The STRUCTURE v2.3 individual assignments of 774 swordfish from 18 localities using the no admixture and correlated allele model and the location prior with A) only the CaM locus, B) all loci except the CaM locus, and C) all loci including the CaM locus.
(TIF)

S3 Fig Genetic partitioning in relation to the South Atlantic OMZ. The oxygen minimum zone (OMZ) in the South Atlantic swordfish feeding grounds at 100 m depth. The annual mean dissolved oxygen (DO) at 100 m depths in mL L^-1^ are from the World Ocean Atlas 2009 (Garcia et al. 2009)^1^. The OMZ depicts a similar boundary as the posterior probability of membership contour maps in Fig 5. ^1^Garcia HE, Locarnini RA, Boyer TP, Antonov JI, Baranova MM, et al., editors (2010) World Ocean Atlas 2009, volume 3: dissolved oxygen, apparent oxygen utilization, and oxygen saturation. Washington, D.C. 344 p.
(TIF)

S1 Table Allele Frequency Distribution. The allele frequency distribution within the eighteen sampled localities for each of the ten nuclear loci.
(XLS)

S2 Table Locus power of assignment. A consensus power of assignment for each locus using WHICHLOCI v.1.0 between North Atlantic (n = 207) and Mediterranean (n = 142) swordfish inferred in STRUCTURE v2.3.
(XLS)

S3 Table Locus power of assignment for the South Atlantic population. A consensus critical population (South Atlantic) power of assignment for each locus using WHICHLOCI v.1.0 between North Atlantic (n = 207) and South Atlantic (n = 297) swordfish inferred in STRUCTURE v2.3.
(XLS)

S4 Table Structure population posterior probability memberships. The average population posterior probability membership (Q) of 18 localities in the Atlantic and Mediterranean inferred in STRUCTURE v2.3.
(XLS)

S5 Table SNP allelic data. The alleles for the ten loci and all 774 swordfish analyzed in this study. Allelic numbering corresponds to reported GenBank alleles for all loci except ALDB. Allelic numbering for ALDB corresponds to STR amplicon size scoring.
(XLSX)
