# Supplementary figures and images for: Multilocus Bayesian Estimates of Intra-Oceanic Genetic Differentiation, Connectivity, and Admixture in Atlantic Swordfish (Xiphias gladius L.)
Source: PLoS One. 2015 Jun 9;10(6):e0127979. doi: 10.1371/journal.pone.0127979 (PMC4461265; doi:10.1371/journal.pone.0127979)

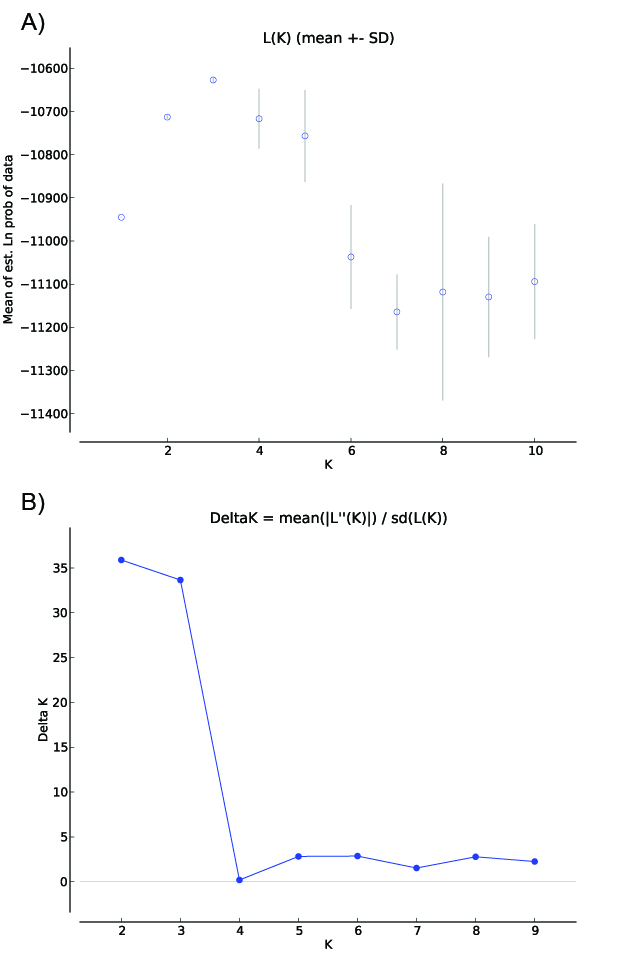

Supplement: S1 Fig — The estimation of the number of clusters (K) in the STRUCTURE v2.3 [55] analysis of Atlantic and Mediterranean swordfish using (A) the ad hoc approach of Prichard et al. [55] and the mean posterior probability of the data (L(K)) and (B) ΔK approach of Evanno et al. [60]. Twenty independent runs for each K value (1–10) were performed using 100,000 MCMC iterations with a burn in period of 100,000. (TIF) [file pone.0127979.s001.tif]

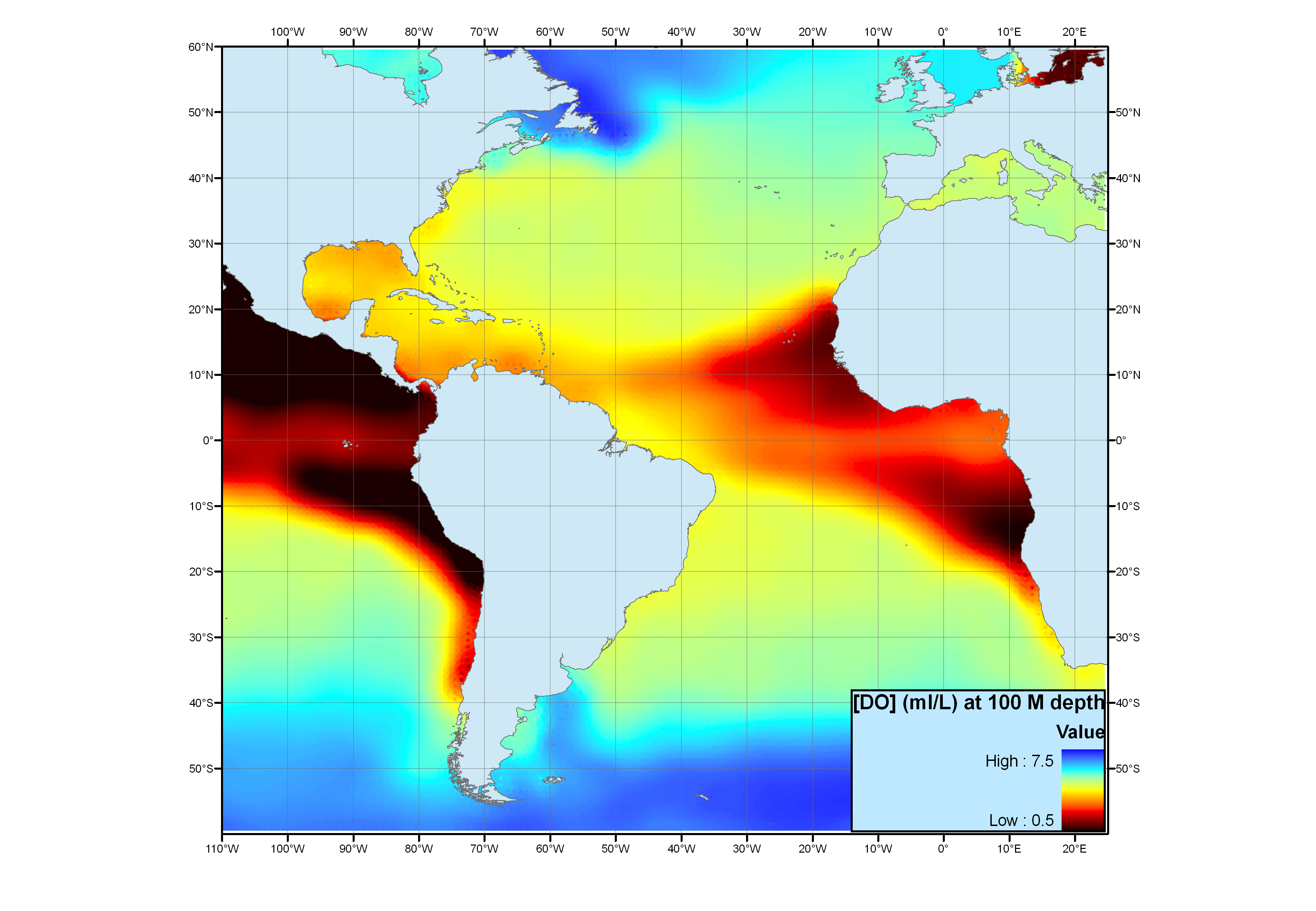

Supplement: S3 Fig — The oxygen minimum zone (OMZ) in the South Atlantic swordfish feeding grounds at 100 m depth. The annual mean dissolved oxygen (DO) at 100 m depths in mL L-1 are from the World Ocean Atlas 2009 (Garcia et al. 2009)1. The OMZ depicts a similar boundary as the posterior probability of membership contour maps in Fig 5. 1Garcia HE, Locarnini RA, Boyer TP, Antonov JI, Baranova MM, et al., editors (2010) World Ocean Atlas 2009, volume 3: dissolved oxygen, apparent oxygen utilization, and oxygen saturation. Washington, D.C. 344 p. (TIF) [file pone.0127979.s003.tif]
